# Regulating Oxygen‐Mediated Reconstruction during Cooling: A New Design Principle for High‐Capacity Sodium Manganese Oxide Cathodes

**DOI:** 10.1002/advs.78064

**Published:** 2026-10-06

**Authors:** Seungmin Lee, Daseul Han, Jae‐Ho Park, Eunji Kwon, Elang Barruna, Sang Hyuk Gong, Mingony Kim, Yiseul Yoo, Seungho Yu, Kyung Yoon Chung, Kyung‐Wan Nam, Se Young Kim, Hyung‐Seok Kim

**Affiliations:** ^1^ Chemistry Division Brookhaven National Laboratory Upton New York USA; ^2^ Department of Energy & Materials Engineering Dongguk University Seoul Republic of Korea; ^3^ Energy Storage Research Center Korea Institute of Science and Technology Seoul Republic of Korea; ^4^ Department of Chemical and Biological Engineering Korea University Seoul Republic of Korea; ^5^ Division of Energy & Environment Technology KIST School Korea University of Science and Technology Seoul Republic of Korea; ^6^ Department of Materials Science and Engineering Korea University Seoul Republic of Korea; ^7^ Yonsei‐KIST Convergence Research Institute Yonsei University Seoul Republic of Korea

**Keywords:** cooling‐rate control, oxygen‐redox cathodes, sodium‐ion batteries, solid‐state synthesis, stacking faults

## Abstract

Deciphering the synthetic origin of oxygen‐redox stability is critical to the design of high‐capacity sodium‐ion cathodes. Although prior studies have correlated electrochemical behavior with pre‐formed structural motifs, the pathways by which these motifs emerge during synthesis remain unclear. Here, we track the structural evolution of layered sodium manganese oxide during solid‐state synthesis using time‐resolved X‐ray diffraction coupled with a real‐time micro‐furnace. We show that oxygen uptake and Mn oxidation occur predominantly during cooling, triggering oxygen‐mediated reconstruction into a framework enriched with manganese vacancies (□Mn) and stacking faults. By kinetically regulating the cooling process, we reveal that slow cooling promotes a spatially homogeneous stacking‐fault‐rich architecture that suppresses in‐plane Mn migration and mitigates irreversible O_2_ release. High‐resolution microscopy directly visualizes the development of stacking faults, while density functional theory shows that these defects substantially increase the barrier for Mn migration. Together, these findings identify cooling‐driven oxygen‐mediated reconstruction as a previously underappreciated synthetic lever for defect engineering and establish a general design principle for stabilizing oxygen redox in layered sodium cathodes.

## Graphical abstract

The cooling process during the solid‐state synthesis of sodium manganese oxides governs oxygen‐mediated reconstruction. Slow cooling promotes a stacking‐fault‐rich framework that suppresses Mn migration, mitigates O_2_ release, and stabilizes oxygen redox with reduced voltage hysteresis. In contrast, quenching yields an SF‐poor structure with incomplete reconstruction, leading to unstable oxygen redox and rapid electrochemical degradation.

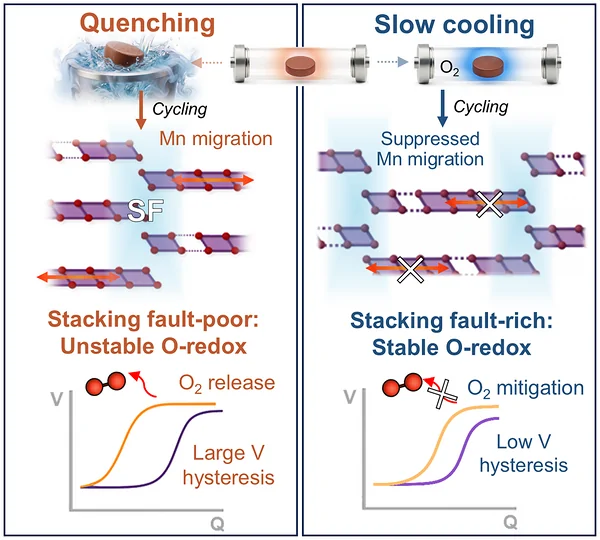

## 1 Introduction

The increasing demand for large‐scale energy storage has intensified interest in sodium‐ion batteries (SIBs) as cost‐effective alternatives to lithium‐based systems [1]. Among the promising cathode chemistries, layered sodium manganese oxides (Na_x_MnO_2_, NMOs) are particularly attractive because of their compositional simplicity, low cost, and earth abundance. However, their practical energy density remains limited by relatively modest reversible capacities, typically below 150 mAh g^−1^ [2, 3]. To overcome this limitation, oxygen redox (O‐redox), in which lattice oxygen participates in charge compensation, has emerged as an effective strategy for accessing additional capacity [4, 5, 6, 7].

Unlike Na‐rich 4d and 5d systems, such as Na_2_RuO_3_ and Na_2_IrO_3_, where O‐redox is stabilized by well‐defined A–O–A configurations (A = alkali metal), Na‐deficient manganese‐based layered oxides generally lack such intrinsic structural motifs because of the large size mismatch between Na^+^ and Mn cations [5, 8]. As a result, O‐redox activity in NMOs is typically enabled through aliovalent substitution or the deliberate introduction of manganese vacancies (□_Mn_), both of which promote the formation of non‐bonding O 2p states [7, 9, 10, 11, 12, 13, 14, 15]. Yet activating O‐redox is only part of the challenge; achieving reversible O‐redox requires structural motifs that suppress irreversible oxygen reorganization, cation migration, and O_2_ release.

The stability of O‐redox depends on two key structural features: (i) the in‐plane distribution of □_Mn_ or A' (substituents) in the MnO_6_ layer [5, 16, 17, 18], and (ii) the stacking nature of MnO_6_ layers [19, 20, 21]. The former governs the distance between lattice oxygens and the energy barrier for cation migration. Upon oxidation, oxidized oxygen ions tend to approach each other, leading to O─O dimerization as a stabilization mechanism [5, 22]. However, excessively shortened O–O distances promote irreversible oxygen loss (O_2_ evolution) and structural degradation. Moreover, when □_Mn_ (or A') are closely clustered, Mn atoms (or A') can more readily migrate in‐plane due to reduced migration barriers, which further drives □_Mn_ (or A') clustering and accelerates unstable O‐redox reactions [23]. Such in‐plane arrangements have been categorized into honeycomb, ribbon, and mesh motifs, with increasing vacancy dispersity corresponding to enhanced O‐redox stability [5, 24, 25]. The second factor, the stacking nature of MnO_6_ layers, has recently been recognized as another crucial parameter governing O‐redox stability alongside in‐plane ordering [19, 20, 21, 26]. Beyond in‐plane ordering, the stacking sequence of MnO_6_ layers and the *c*‐axis registry of vacancies define the local topology of □_Mn_–O–□_Mn_ environments, thereby governing cation migration pathways and oxygen‐redox stability. These structural characteristics regulate cation migration pathways and local bond geometry in □_Mn_–O–□_Mn_ (or A–O–A) configurations [21]. Furthermore, stacking faults (SFs) modify local A–O–A geometry, thereby influencing O‐redox activity [20].

Despite recent progress in understanding how structural motifs, including in‐plane □_Mn_ (or A') ordering and layer stacking, influence O‐redox stability, the synthetic origin of these motifs remains largely unexplored. Given that early‐stage structural instabilities such as cation migration, vacancy clustering, and O–O dimerization can trigger irreversible degradation during electrochemical cycling [27], clarifying their synthetic origin is essential for the rational design of intrinsically stable O‐redox NMO cathodes.

To address this fundamental question, we investigate the structural evolution of O‐redox‐active NMOs during solid‐state synthesis and its impact on O‐redox stability. As a model system, we focus on mesh‐type Na_2_Mn_3_O_7_ (Na_4/7_Mn_6/7_□_Mn(1/7)_O_2_). It exhibits well‐defined √7 × √7 in‐plane □_Mn_ ordering and the presence of stacking faults (SFs), both of which have been closely linked to enhanced O‐redox reversibility (voltage hysteresis < 50 mV) [6, 28, 29, 30, 31, 32, 33, 34, 35, 36]. This combination of ordered vacancies and intrinsic stacking disorder provides a unique platform to examine how such structural motifs emerge during synthesis and cooperatively stabilize O‐redox chemistry. Previous studies suggest that the formation of □_Mn_ in NMOs is driven by oxygen uptake during the cooling region, which is thermodynamically favorable near or below 600°C [37, 38, 39, 40, 41, 42, 43, 44, 45, 46]. In this process, excess oxygen oxidizes Mn^3+^ to Mn^4+^, thereby generating vacancies in the MnO_6_ layers, as described by Equation (1):

(1) $$NaxMnO2+y/2O2\;\to Nax\left[Mn□Mn\right]O2+y$$

This view suggests that _Mn_ ordering is not an intrinsic attribute of the high‐temperature parent phase, but rather the consequence of a cooling‐driven oxygen‐mediated reconstruction process. Such oxygen‐driven reconstruction has been widely observed in Mn‐based oxides and is known to be highly sensitive to thermal history [42, 47, 48]. Building on this understanding, we propose that the cooling rate during synthesis serves as a decisive synthetic parameter in regulating the extent of structural reconstruction, including □_Mn_ formation and stacking faults. To test this hypothesis, we systematically control the cooling rate during the synthesis of Na_2_Mn_3_O_7_ and track its structural evolution using time‐resolved and ex‐situ characterization techniques.

## 2 Results and Discussion

### 2.1 Cooling‐Dependent Structural Differences and Electrochemical Reversibility

To regulate oxygen uptake during synthesis, the post‐annealing cooling rate was varied. A precursor mixture of NaNO_3_ and MnCO_3_ was heated at 600°C for 4 h and either quenched in liquid N_2_ (NMO‐Q) or slowly cooled at 1.8°C min^−1^ (NMO‐S) (Figure 1a, synthesis details are provided in the Supporting Information). The long‐range structures were examined using synchrotron X‐ray diffraction (SXRD, Figure 1b). Phase fractions and lattice parameters obtained from profile matching are summarized in Tables S1 and S2, with representative fitting results shown in Figure S1. Both samples were composed predominantly of P′3‐type Na_2_Mn_3_O_7_ (′: monoclinic distortion; space group: P $\bar{1}$), together with a minor fraction of P3‐type Na_x_MnO_2_ (space group: R3m). Profile matching yielded R_wp_ values of 7.19% and 7.68% for NMO‐S and NMO‐Q, respectively. Characteristic reflections of the P′3 phase, such as those at 1.71, 3.42, 3.88, and 3.98°, were observed in both samples, consistent with stacking‐fault‐related features of the [Mn_3_O_7_]^2−^ layers [31, 32]. Notably, NMO‐S exhibited a higher fraction of the P′3 phase (98.3%) and a lower fraction of the P3 phase (1.7%) than NMO‐Q (90.1% P′3 and 9.9% P3; inset table in Figure 1b). In contrast, NMO‐Q displayed sharper reflections at 4.22° and 4.64°, corresponding to the (012) and (104) planes of the P3 phase, respectively, together with an additional peak at 2.38° assigned to the (20$\bar{1}$) plane, a superstructure reflection characteristic of P3‐type NMOs [4, 18, 21, 49].

**FIGURE 1:**
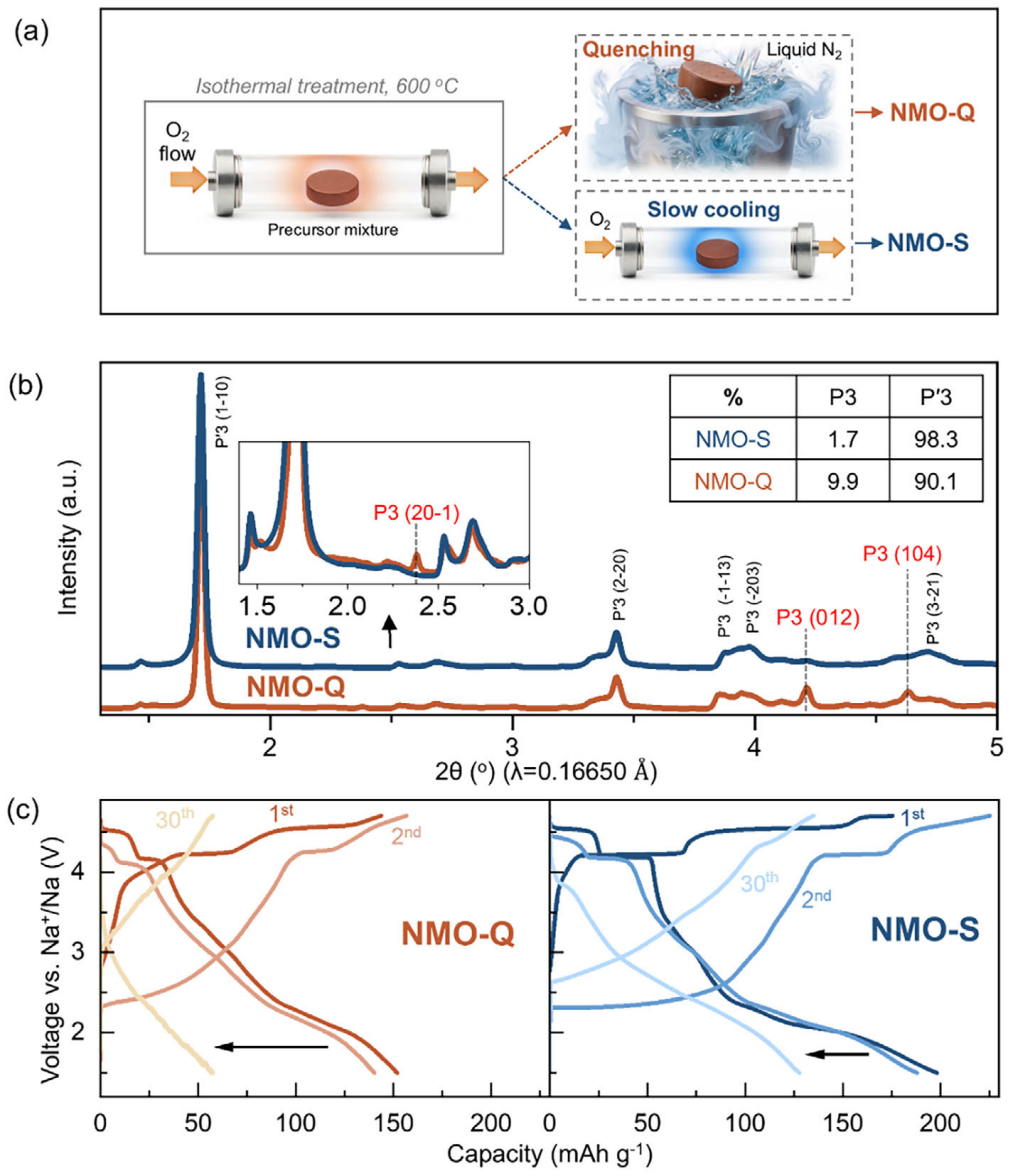
Structural characterization of NMOs with different cooling rates. (a) Schematic illustration of the solid‐state synthesis procedure for NMOs with different cooling rates. NMO‐Q was obtained by quenching the pellet in liquid N_2_ immediately after isothermal treatment, while NMO‐S was cooled at a rate of 1.8°C min^−1^. (b) Synchrotron XRD patterns of NMOs (inset: enlarged XRD pattern view; table: phase fractions obtained from profile matching). (c) Charge–discharge profiles of NMOs at the first, second, and 30^th^ cycles within 1.5–4.7 V_Na+/Na_.

To evaluate the impact of these subtle structural differences on electrochemical performance, galvanostatic charge/discharge tests were conducted at 7.5 mA g^−1^ over 1.5–4.7 V vs Na^+^/Na, a voltage window involving both cationic and oxygen redox processes (Figure 1c). The first charge capacities were 175 and 143 mAh g^−1^ for NMO‐S and NMO‐Q, respectively, with NMO‐S showing a more extended plateau above 4.0 V, corresponding to the O‐redox region. Despite this extensive O‐redox activity, NMO‐S delivered a higher discharge capacity than NMO‐Q (198 vs. 127 mAh g^−1^). Upon further cycling, both cationic and O‐redox contributions decayed rapidly in NMO‐Q, which retained only 37% of its capacity by the 30th cycle, whereas they were better maintained in NMO‐S, which retained 64% (Figures S2 and S3).

These results highlight the critical importance of initial structural stability in O‐redox cathodes. Because both samples exhibited comparable particle sizes (50–100 nm; Figure S4), the observed differences in electrochemical performance are attributed primarily to cooling‐dependent variations in phase composition and structural defects rather than particle‐size effects. Given the strong sensitivity of O‐redox chemistry to local vacancy topology and stacking structure, even modest differences in phase composition and stacking characteristics can lead to pronounced changes in electrochemical reversibility.

### 2.2 Impact of Initial Structure on O‐Redox Stability

To more directly probe how the initial structure influences O‐redox reversibility, the voltage window was narrowed to the O‐redox region (4.5 – 3.5 V). In the first cycle, NMO‐S exhibited a lower total charge capacity (64.1 mAh g^−1^) compared to NMO‐Q (92.8 mAh g^−1^) (Figure 2a). However, the charge in NMO‐S was dominated by O‐redox above 4.25 V (82.8% of the total capacity), whereas NMO‐Q showed a larger contribution from Mn‐related redox below 4.25 V (54.8% of the total capacity) [28]. Despite its lower total charge capacity, NMO‐S delivered a higher initial Coulombic efficiency after discharge (52.9% vs. 34.3% for NMO‐Q). The dQ/dV profiles further revealed substantially lower voltage hysteresis for NMO‐S (ΔV = 29 mV) than for NMO‐Q (ΔV = 74 mV). Importantly, in situ differential electrochemical mass spectrometry (DEMS; Figure 2b) detected O_2_ evolution from NMO‐Q during the first charge, whereas no gas evolution was observed for NMO‐S, indicating markedly greater oxygen‐redox reversibility. These results suggest that the cooling‐reconstructed structure of NMO‐S better stabilizes the local Mn–O framework associated with high‐voltage O‐redox, thereby suppressing irreversible oxygen release and mitigating structural degradation during the initial charge [28].

**FIGURE 2:**
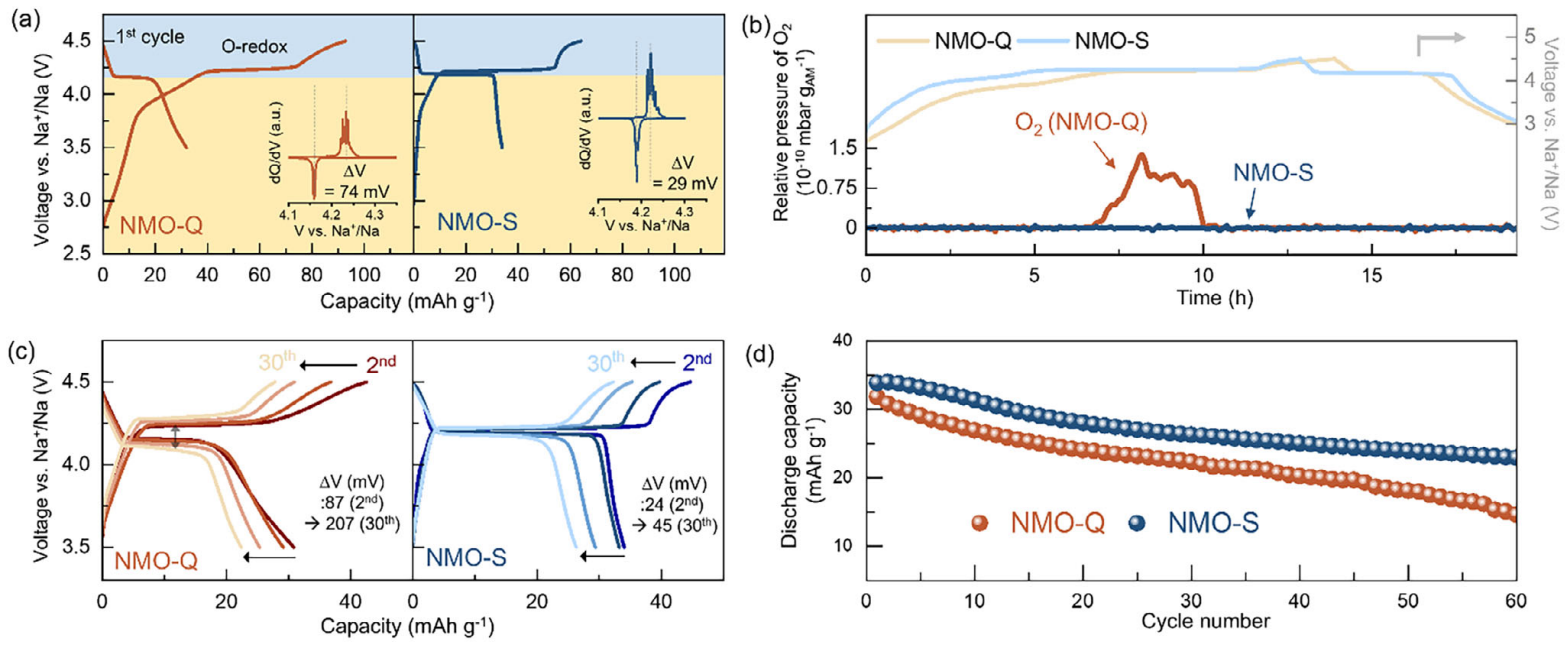
Electrochemical evaluation of O‐redox stability of NMOs. (a) Charge/discharge profiles during the initial cycle. (b) In situ differential electrochemical mass spectrometry (DEMS) analysis during the first cycle. (c) Charge/discharge profiles at the second, fifth, 15th, and 30th cycles. (d) Cycling performance of NMOs. Galvanostatic charge/discharge measurements were performed at 7.5 mA g^−1^ within a voltage window of 4.5−3.5 V_Na+/Na_.

Extended cycling further confirmed the superior O‐redox stability of NMO‐S (Figure 2c). By the 30^th^ cycle, NMO‐S retained 87.2% of its O‐redox charge capacity, compared with 79.7% for NMO‐Q, while maintaining much lower voltage hysteresis (ΔV = 45.2 vs. 207 mV, respectively). These electrochemical trends were consistently reproduced across three independently assembled cells for each material (Figure S5). These results indicate that NMO‐S more effectively resists degradation processes associated with Mn migration and O_2_ loss. Consistently, NMO‐S retained 68% of its capacity after 60 cycles, whereas NMO‐Q retained only 46% (Figure 2d and Figure S6). The superior robustness of NMO‐S was also evident across multiple voltage windows (4.3–3.5 and 4.7–3.5 V vs. Na^+^/Na), under which it consistently outperformed NMO‐Q (Figures S7–S9). To directly compare their rate performance, NMO‐Q and NMO‐S were tested under identical conditions at various current densities (Figure S10). NMO‐S retained 71% of its average discharge capacity at 150 mA g^−1^ relative to that at 7.5 mA g^−1^, compared with 54% for NMO‐Q. This indicates that its stacking‐fault‐rich framework does not compromise high‐rate oxygen‐redox performance. Combined GITT–EIS/DRT analysis suggests that the improved rate retention of NMO‐S originates primarily from reduced diffusion limitation at low SoC, whereas both samples exhibit comparable diffusion kinetics in the high‐voltage O‐redox region (Figures S11 and S12). The greater low‐SoC resistance of NMO‐Q may reflect local structural rearrangements in its P3‐derived framework.

Taken together, these results show that cooling‐dependent structural differences exert a disproportionate influence on the reversibility and long‐term stability of O‐redox chemistry. To identify when and how these structural differences emerge, we next track the real‐time phase evolution of NMOs during solid‐state synthesis using time‐resolved X‐ray diffraction (TR‐XRD) coupled with a real‐time X‐ray analytical micro‐furnace (RT‐XAMF) [50, 51].

### 2.3 Establishment of the High‐Temperature Parent Framework

To monitor phase evolution during solid‐state synthesis, the precursor mixture of NaNO_3_ and MnCO_3_ was heated to 600°C and held for 4 h in a real‐time X‐ray analytical micro‐furnace (RT‐XAMF). Time‐resolved X‐ray diffraction (TR‐XRD) patterns were collected continuously throughout the heating and isothermal stages (Figure 3a), while concurrent thermogravimetric analysis (TGA) tracked the corresponding weight changes (Figure 3b). As the temperature increased, sequential decomposition of NaNO_3_ and MnCO_3_ was observed (line plots in Figure S13) [37]. Around 350°C, the appearance of the (003) reflection near 15.5° indicated the formation of a layered P3‐type Na_x_MnO2 phase (space group: R3m) [37, 52]. Topotactic sodiation then progressed with increasing temperature, as evidenced by the growth of characteristic P3 reflections at 31.0, 35.6, 41.2, 44.2, and 51.6°, corresponding to the (006), (101), (012), (104), and (015) planes, respectively [37, 52, 53, 54, 55, 56]. Notably, no reflections corresponding to the final P′3‐type Na_2_Mn_3_O_7_ phase were detected during the heating or isothermal stages (line plots in Figure S14), indicating that the target framework was not established at the high‐temperature stage.

**FIGURE 3:**
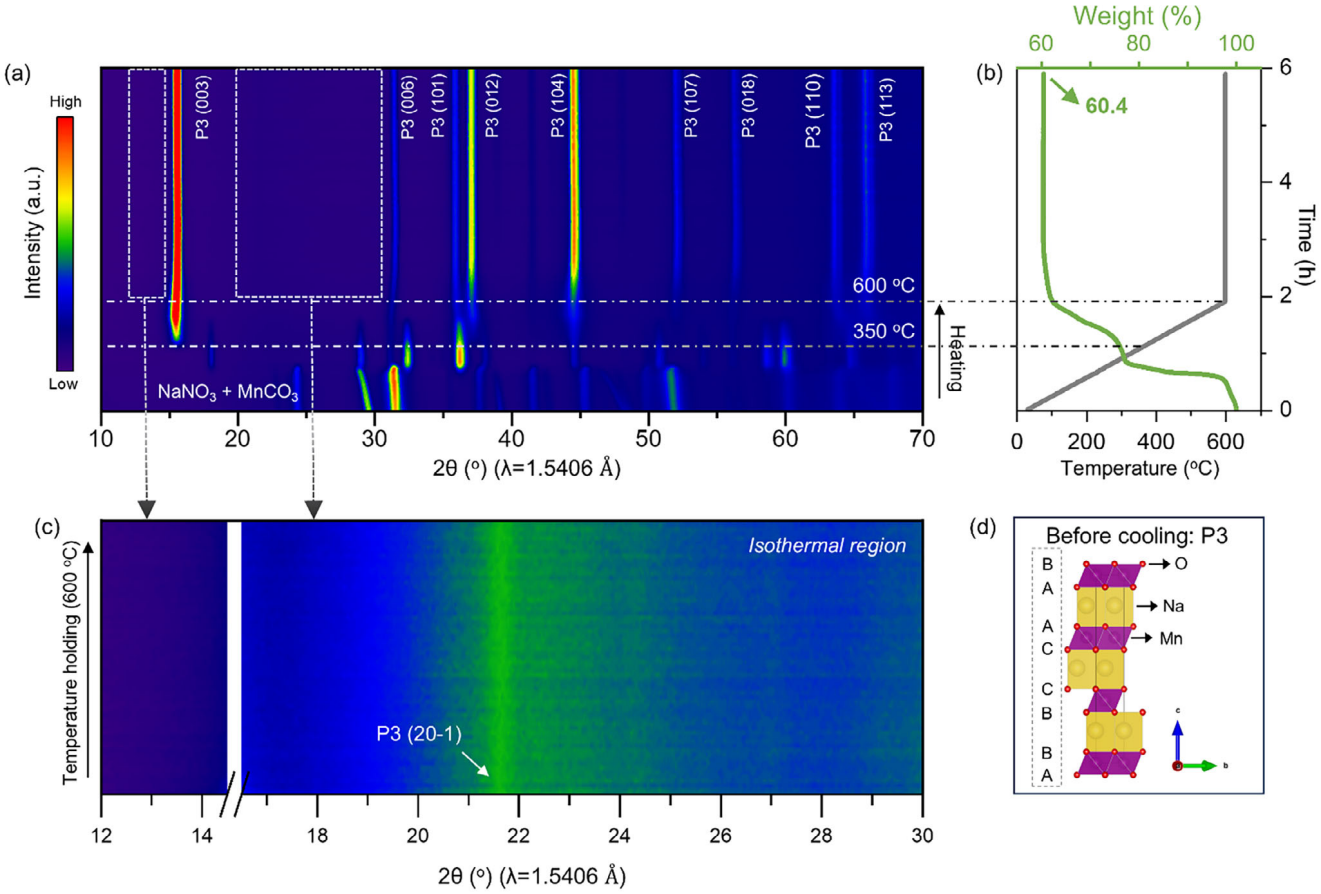
Time‐resolved X‐ray diffraction (TR‐XRD) patterns during synthesis of NMOs. (a) Contour plots of XRD patterns with time during heating and isothermal treatment of precursor mixture (NaNO_3_ and MnCO_3_) with (b) corresponding thermogravimetric analysis (TGA); The mixture of NaNO_3_ and MnCO_3_ (2:3 in molar ratio) was heated at 600°C for 4 h at a heating rate of 5°C min^−1^. (c) Enlarged contour plots of (a) in the range of 12.0 − 14.5 ^o^ and 16.5 − 30.0 ^o^. (d) Structure image of P3‐Na_x_MnO_2_ at the end of isothermal treatment.

A broad reflection at ∼21.5° also emerged during isothermal treatment (Figure 3c), corresponding to the (20$\bar{1}$) plane and indicative of the P3 superstructure in layered sodium manganese oxides [4, 18, 21]. This feature indicated coupled cation ordering within the transition‐metal layers of the P3 phase, likely associated with periodic Mn^3+^/Mn^4+^ ordering [5]. Crucially, superstructure reflections associated with √7 × √7 _Mn_ ordering, typically observed near ∼13.8° and in the 20–30° range, were absent at this stage, showing that _Mn_ formation and structural reconstruction had not yet occurred. As illustrated in Figure 3d, the structure at the end of isothermal treatment therefore remained a P3‐type framework with limited monoclinic distortion and without long‐range □_Mn_ ordering. Together, these results show that the final O‐redox‐active reconstructed framework was established only during subsequent cooling.

### 2.4 Cooling‐Driven Structural Reconstruction

The decisive reconstruction process was captured during cooling, as shown in Figure 4a. To maximize oxygen uptake during cooling, the cooling rate was reduced to 1.8°C min^−1^ (NMO‐S; consistent with Figures 1 and 2). The diffraction patterns collected during cooling revealed two distinct temperature regimes: (i) 600°C–520°C and (ii) below 520°C (line plots in Figure S15). In the higher‐temperature regime, P3‐type reflections such as (003) gradually shifted toward higher 2θ values, indicating a decrease in d‐spacing. This shift was consistent with Mn^3+^ → Mn^4+^ oxidation, as described by Equation 1 [57]. Concurrent TGA provided direct support for this interpretation, showing a significant weight increase of 5.05 wt.% above 500°C due to oxygen uptake (Figure 4b) [40, 41, 48]. As cooling proceeded, the P3 reflections, including (101), (012), and (015), began to broaden and split (Figure S15), marking the onset of structural transformation. These evolving features were assigned to the P′3 phase, indicating monoclinic distortion of the parent P3 framework [46, 58, 59, 60]. This transformation was further corroborated in the superstructure regions (13.0–14.5° and 16.5–30.0°; Figure S16). Notably, the broad peak at ∼21.5°, characteristic of the P3 superstructure, diminished near 520°C. Crucially, new reflections associated with in‐plane □_Mn_ ordering, characteristic of the P′3 phase, emerged as the P3 superstructure weakened, indicating the formation of □_Mn_ in the transition‐metal layers. An independent TR‐XRD measurement using a separately prepared NMO‐S precursor batch reproduced the same phase evolution and cooling‐induced structural reconstruction, confirming the reproducibility of these observations (Figure S17).

**FIGURE 4:**
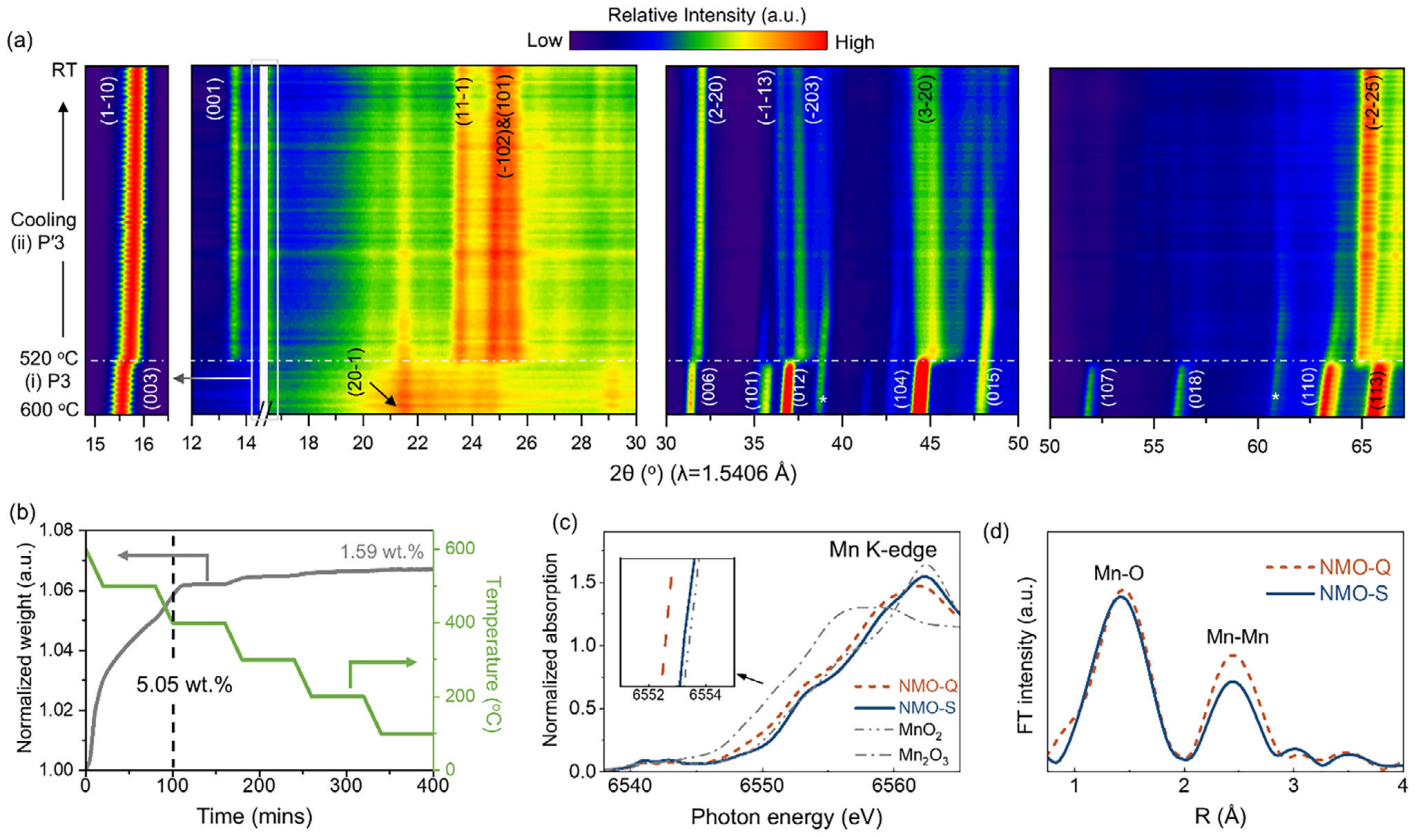
Time‐resolved X‐ray diffraction (TR‐XRD) patterns during synthesis of NMOs (a) Contour plots of XRD patterns with times during cooling process (cooling rate: 1.8°C min^−1^, named as NMO‐S in this work); *: O′3. (b) Corresponding TGA results (normalized weight of NMOs at the end of the isothermal region is set to 1); (c) Mn K‐edge XANES of NMOs; (d) EXAFS spectra of NMOs.

Mn K‐edge XAS further supported this cooling‐induced reconstruction: NMO‐S showed a higher average Mn oxidation state (3.89; Mn^4+^/Mn^3+^ = 8.10) than NMO‐Q (3.77; Mn^4+^/Mn^3+^ = 3.35) (Figure 4c), confirming a greater extent of Mn oxidation driven by oxygen uptake. EXAFS analysis likewise indicated a higher □_Mn_ concentration in NMO‐S, as evidenced by the reduced Fourier‐transform intensity of the Mn–Mn shell (Figure 4d). Consistent with this interpretation, TEM and X‐ray pair distribution function (PDF) analyses (Figure S18) supported vacancy formation in the transition‐metal layers, with the reduced Mn–Mn correlation at 2.88 Å in NMO‐S providing additional evidence for increased □_Mn_ concentration. This suggests a sequential reconstruction pathway in which oxygen uptake during cooling drives Mn oxidation and □_Mn_ formation, which in turn promotes SF development and ultimately stabilizes O‐redox chemistry. Importantly, a sample prepared at an intermediate cooling rate (9.0°C min^−1^; Figures S19–S21) exhibited intermediate reconstruction behavior, including mixed P3/P′3 superstructure reflections and compromised O‐redox reversibility. The smaller oxygen uptake above 500°C at this cooling rate (2.72 wt.%, compared with 5.05 wt.% for NMO‐S) limited Mn oxidation, resulting in less pronounced □_Mn_ formation and suppressed development of stacking faults. Together, these results identify oxygen uptake above ∼500°C as the driving force for cooling‐induced reconstruction and establish cooling rate as a key synthetic parameter governing vacancy formation, stacking‐fault development, and the resulting O‐redox stability.

HR‐TEM provided direct real‐space evidence for the cooling‐induced reconstruction inferred from TR‐XRD (Figure 5). In NMO‐Q, stacking faults (SFs) were only locally observed within otherwise well‐ordered transition‐metal layers, as highlighted by the white dashed box in Figure 5a. The corresponding FFT pattern (inset) displayed sharp diffraction spots with only faint streaking, consistent with a largely ordered layered structure containing a low density of SFs. In contrast, NMO‐S exhibited a uniformly SF‐rich framework throughout the particle, as indicated by the white arrows in Figure 5c. Its FFT pattern showed pronounced streaks parallel to the stacking direction, confirming a homogeneous distribution of SFs. Enlarged images and the corresponding line profiles (Figure 5b,d) further highlighted the contrast between the two samples: NMO‐Q retained mostly periodic interlayer contrast with only localized disruptions, whereas NMO‐S showed frequent interruptions in periodicity and irregular interlayer spacings consistent with abundant SFs across the particle. Collectively, these observations indicate that slow cooling produced a spatially homogeneous SF‐rich framework in NMO‐S, whereas quenching preserved partially reconstructed P3‐like domains with localized stacking mismatches. This real‐space contrast is fully consistent with the cooling‐induced phase evolution revealed by TR‐XRD and suggests that SFs are not merely a structural byproduct of slow cooling, but a functionally important feature that contributes to O‐redox stabilization.

**FIGURE 5:**
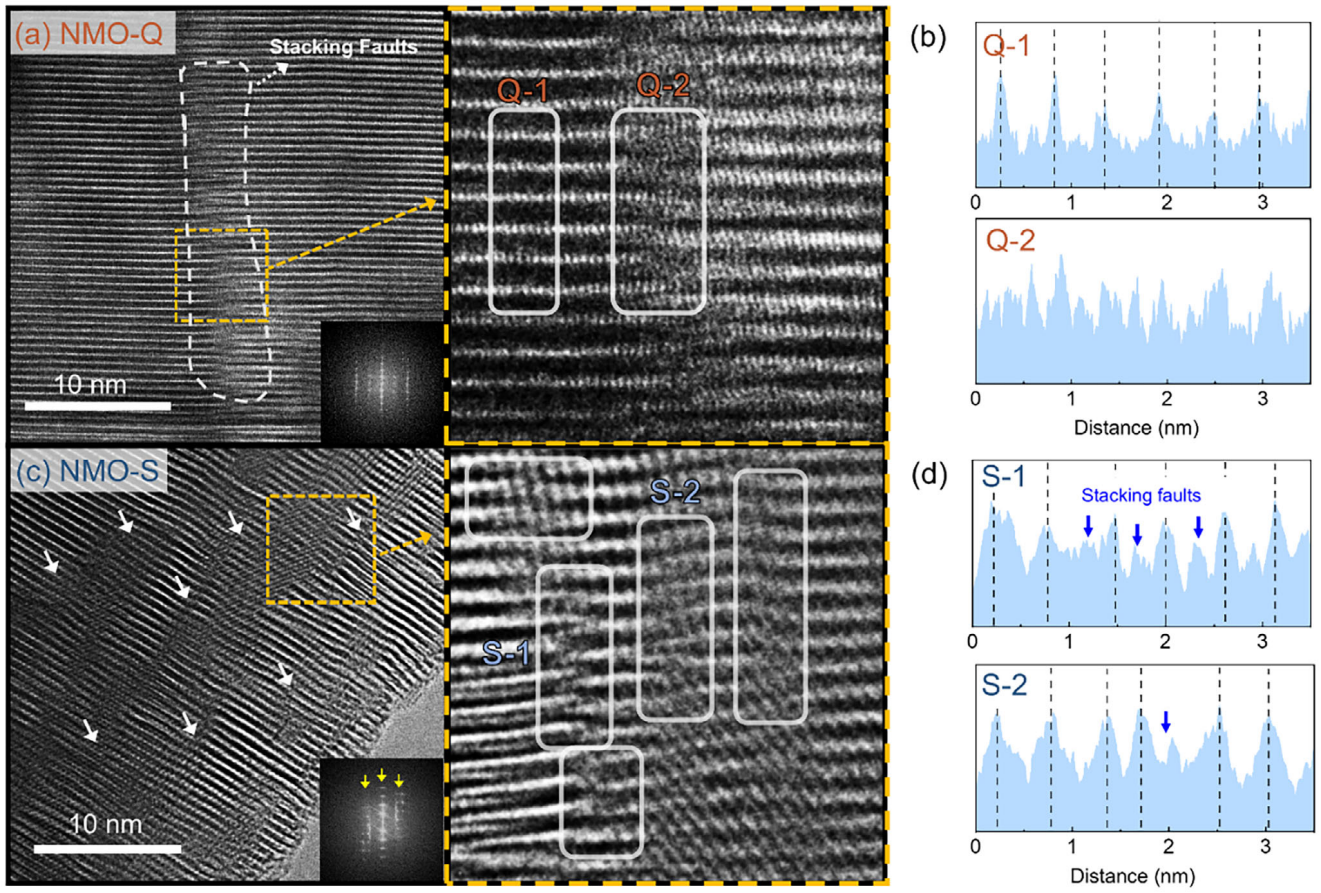
Stacking fault characterization of NMO‐Q and NMO‐S. (a) HR‐TEM image of NMO‐Q showing locally confined stacking faults and the corresponding FFT pattern (inset); (b) Line profiles from regions in (a); (c) HR‐TEM image of NMO‐S with uniformly distributed stacking faults across the particle (white arrows), confirmed by strong streaks in the FFT (inset). (d) Line profiles from regions in (c).

### 2.5 Mechanistic Origin of O‐Redox Stabilization in the SF‐Rich Reconstructed Framework

To clarify the mechanistic role of stacking faults (SFs) in O‐redox stabilization, we performed density functional theory (DFT) calculations (Figure 6a–c). Because in‐plane Mn migration in O‐redox cathodes promotes vacancy clustering and increases the likelihood of O–O bond formation between oxidized oxygen ions [5], we calculated the energy barriers for in‐plane Mn migration in two local environments: an SF‐near region, motivated by the SF‐rich structure of NMO‐S, and a bulk‐like region, motivated by the more ordered local environments in NMO‐Q. In both cases, Mn migration was modeled by evaluating the energy profile for a Mn ion hopping between adjacent octahedral sites (bulk in Figure 6a and SF‐near in Figure 6b). Climbing‐image nudged elastic band (CI‐NEB) calculations revealed that the migration barrier was strongly influenced by the presence of SFs. In the bulk‐like region, Mn migration followed a symmetric and energetically favorable pathway with an activation barrier of 2.49 eV (Figure 6c). By contrast, the SF‐near region exhibited a distorted migration pathway arising from local strain and stabilization of the oxygen framework, resulting in a substantially higher barrier of 3.84 eV. The increase in activation energy (≈1.35 eV) indicates that SFs effectively suppress in‐plane Mn migration and thereby mitigate irreversible structural transformations associated with O‐redox degradation [61, 62]. These theoretical insights are consistent with experimental observations. O K‐edge XANES spectra collected in the pristine state, after the first charge to 4.5 V, and after subsequent discharge showed substantially more reversible oxygen‐related spectral evolution in NMO‐S than in NMO‐Q (Figure S22) [63]. After discharge, the spectrum of NMO‐S recovered more closely toward its pristine profile, whereas NMO‐Q retained more pronounced residual changes, indicating a more reversible evolution of the oxygen‐related electronic structure and local oxygen environment in NMO‐S. Likewise, in situ XRD measurements during the initial cycles showed that the characteristic P′3 reflections, including (1$\bar{1}$0), (2$\bar{2}$0), ($\bar{2}$03), and (3$\bar{2}$0) at ∼ 16, 32, 38.5 and 44.5° respectively, together with the superstructure‐related reflections in the 13.0–14.5° and 16.5–30.0° ranges, were substantially better preserved in NMO‐S than in NMO‐Q (Figures S23 and S24). Sequential Le Bail analysis showed relatively slight volume changes up to approximately 4.5 V, followed by a pronounced contraction at higher voltage. Despite its larger contraction at the highest state of charge, NMO‐S exhibited more complete lattice recovery after discharge, whereas NMO‐Q retained larger residual lattice deviations. Together, these results indicate that SFs raise the energetic cost of Mn migration and stabilize the local oxygen environment, thereby contributing to enhanced O‐redox reversibility.

**FIGURE 6:**
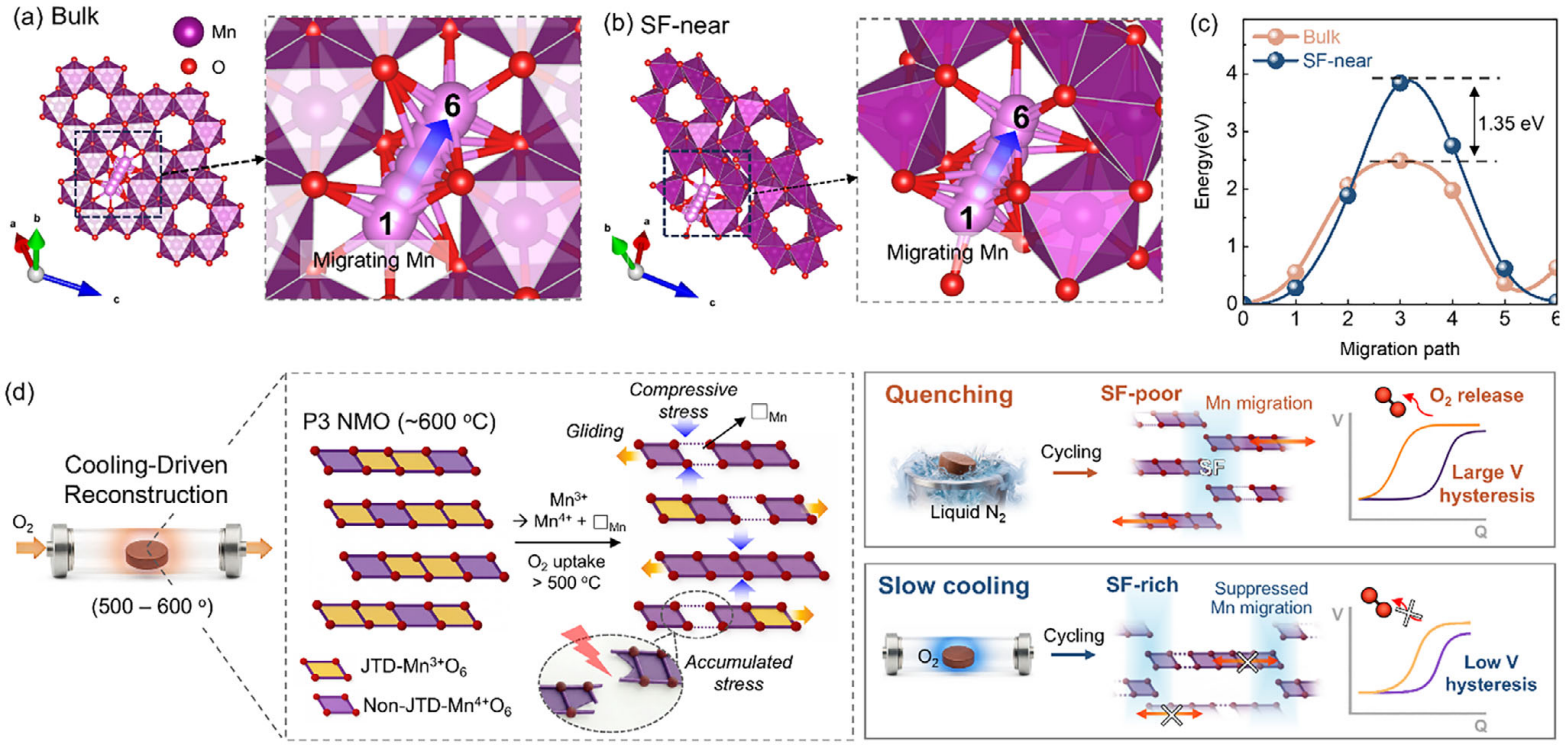
Effect of stacking faults on Mn migration and O‐redox behavior in NMO cathodes. Mn migration pathways between adjacent octahedral sites in (a) bulk and (b) SF‐near regions; (c) In‐plane Mn migration energy barrier of NMOs using DFT‐based climbing image nudged elastic band (CI‐NEB) calculations; (d) Schematic illustration of the cooling‐dependent reconstruction mechanism. Oxygen uptake at high temperature induces Mn oxidation, generating compressive stress in the MnO_6_ layers and promoting layer gliding and stacking‐fault (SF) formation, assisted by Mn vacancies. Rapid cooling (NMO‐Q) freezes an incomplete, SF‐poor framework prone to Mn migration and irreversible O–O dimer formation during cycling. In contrast, slow cooling (NMO‐S) enables stress release via SF‐rich P′3 domains, suppressing Mn migration and enhancing O‐redox stability despite only a subtle difference in P′3 fraction.

Building on this mechanistic understanding, we propose a cooling‐driven reconstruction mechanism for O‐redox‐active NMO cathodes (Figure 6d). During cooling above ∼500°C, oxygen uptake drives Mn^3+^ → Mn^4+^ oxidation, which is accompanied by contraction of the MnO_6_ framework. This contraction generates in‐plane compressive stress within the MnO_6_ layers. Together with the reduced Mn–O–Mn connectivity arising from □_Mn_ formation, this stress promotes the development of SFs, which relieve strain and stabilize the structure during slow cooling (NMO‐S). As a result, the SF‐rich framework raises the barrier for Mn migration, thereby suppressing vacancy clustering and O–O dimer formation. In contrast, rapid cooling limits oxygen uptake and yields incomplete reconstruction, leaving residual P3 domains and a lower density of SFs. These findings are consistent with the enhanced structural reversibility and O‐redox stability observed in NMO‐S. Notably, although the difference in P′3 phase fraction is only ∼8.2%, the improvement in O‐redox stability is substantial, underscoring the disproportionate stabilizing effect of SFs. The practical applicability of NMO‐S was further evaluated in full cells using pre‐sodiated hard carbon as the anode (Figure S25). During the first cycle, the NMO‐S full cell delivered a higher discharge capacity of 188 mAh g^−1^ than the NMO‐Q full cell (148 mAh g^−1^), providing preliminary evidence that the enhanced O‐redox stability of the NMO‐S framework is retained beyond Na‐metal half‐cell configurations.

## 3 Conclusion

In summary, we elucidate a synthesis‐driven reconstruction mechanism in O‐redox‐active sodium manganese oxides and establish its direct link to oxygen‐redox stability. By systematically tuning the cooling process, we show that oxygen uptake during cooling drives structural reconstruction toward a uniform, SF‐rich P′3 framework with enhanced □_Mn_ formation, whereas rapid cooling results in incomplete reconstruction with residual P3 domains and an SF‐poor structure. This cooling‐induced defect architecture raises the barrier for Mn migration and thereby suppresses the irreversible structural evolution associated with O‐redox degradation. Supported by TR‐XRD, HR‐TEM, XAS, and DFT analyses, these findings establish cooling‐rate‐controlled defect engineering as an effective synthetic strategy and a broader design principle for stabilizing oxygen redox in high‐voltage sodium layered cathodes.

## Author Contributions

**Seungmin Lee**: conceptualization, methodology, investigation, validation, formal analysis, visualization, writing – original draft, writing – review and editing, data curation. **Daseul Han**: investigation, formal analysis, data curation. **Jae‐Ho Park**: investigation, data curation. **Eunji Kwon**: investigation, software, formal analysis, writing – review and editing, visualization. **Elang Barruna**: investigation. **Sang Hyuk Gong**: investigation. **Mingony Kim**: investigation, data curation. **Yiseul Yoo**: investigation, data curation. **Seungho Yu**: methodology, resources, supervision, writing – review and editing, software. **Kyung Yoon Chung**: supervision, resources, methodology. **Kyung‐Wan Nam**: supervision, methodology, resources. **Se Young Kim**: writing – review and editing, conceptualization, validation, supervision. **Hyung‐Seok Kim**: writing – review and editing, conceptualization, validation, project administration, resources, supervision, funding acquisition, formal analysis.

## Conflicts of Interest

The authors declare no conflicts of interest.

## Supporting information




**Supporting File**: advs78064‐sup‐0001‐SuppMat.docx.

## Data Availability

The data that support the findings of this study are available from the corresponding author upon reasonable request.
