# Supplementary material for: Regulating Oxygen‐Mediated Reconstruction during Cooling: A New Design Principle for High‐Capacity Sodium Manganese Oxide Cathodes
Source: Adv Sci (Weinh). 2026 Oct 6;13(58):e78064. doi: 10.1002/advs.78064 (PMC13643103; doi:10.1002/advs.78064)
Supplement: Supplementary file 1 [file ADVS-13-e78064-s001.docx]

***Supporting information***

of

Regulating Oxygen-Mediated Reconstruction During Cooling: A New Design Principle for High-Capacity Sodium Manganese Oxide Cathodes

Seungmin Lee^a^*, Daseul Han^b^, Jae-Ho Park^c^, Eunji Kwon^c,d^, Elang Barruna^c,e^,

Sang Hyuk Gong^c^, Mingony Kim^c,e^, Yiseul Yoo^c,f^, Seungho Yu^c,e^, Kyung Yoon Chung^c,e^, Kyung-Wan Nam^b^, Se Young Kim^c^*, and Hyung-Seok Kim^c,e,g^*

*^a^Chemistry Division, Brookhaven National Laboratory, Upton, 11973, New York, United States*

*^b^Department of Energy & Materials Engineering, Dongguk University, Seoul, 04620, Republic of Korea*

*^c^Energy Storage Research Center, Korea Institute of Science and Technology, Seoul 02792, Republic of Korea*

*^d^Department of Chemical and Biological Engineering, Korea University, Seoul, Republic of Korea*

*^e^Division of Energy & Environment Technology, KIST School, Korea University of Science and Technology, Seoul 02792, Republic of Korea*

*^f^Department of Materials Science and Engineering, Korea University, 145, Anam-ro, Seongbuk-gu, Seoul, 02841, Republic of Korea*

*^g^Yonsei-KIST Convergence Research Institute, Yonsei University, 50 Yonsei-ro, Seodaemun-gu, Seoul, 03722, Republic of Korea*

*Corresponding Authors: Seungmin Lee, Se Young Kim, and Hyung-Seok Kim

**Experimental details**

*Solid-state synthesis of sodium manganese oxides (NMOs)*

NaNO_3_ (99.0%, DAEJUNG) and MnCO_3_ (99.9%, Sigma-Aldrich) were mixed in a molar ratio of 2:3 using a high-energy ball mill (stainless-steel jars and balls, Ar-filled; ball-to-powder ratio = 10) for 1 h. The mixture was pelletized and heated at 600 ^o^C for 4 h in a tube furnace under flowing O_2_ (heating rate: 5 ^o^C min^-1^). After heating, the samples were quenched using liquid N_2_ (NMO-Q) and cooled down with rates of 1.8 ^o^C min^-1^ (NMO-S), respectively.

*Time resolved X-ray diffraction (TR-XRD) measurement equipped with real-time X-ray analytical micro-furnace (RT-XAMF)*

TR-XRD measurements using a real-time X-ray analytical micro-furnace (RT-XAMF) followed previously reported procedures.^[1,2]^ The as-prepared precursor mixture was loaded into quartz capillaries and mounted on a thermal stage on an X-ray diffractometer (R-AXIS IV, *Rigaku Corp*.). Samples were heated to 600 °C at 5 ^o^C min^-1^ with a 4 h isothermal hold, matching bulk synthesis conditions. Diffraction patterns were collected in transmission geometry using a Rigaku R-AXIS IV imaging plate detector with Mo Kα radiation (λ = 0.7107 Å). For NMO-S, diffraction patterns were also recorded continuously during the subsequent cooling step at 1.8 ^o^C min^-1^ to monitor structural evolution during reconstruction. Two-dimensional images were integrated to obtain one-dimensional patterns.

*Electrochemical evaluation of O-redox cathodes*

NMO powder, Super P, and PVDF binder (8:1:1 by weight) were dispersed in NMP and cast onto Al foil, then dried at 80 ^o^C under vacuum for 12 h. Circular electrodes (Ø 12 mm) were punched from the dried films. Na||NMO 2032-type coin cells were assembled in an Ar-filled glove box using NMO electrodes (working electrode, Ø 12 mm), glass fiber separator, Na metal (counter/reference electrode, Ø 13 mm), and 1.0 M NaPF_6_ in ethylene carbonate/propylene carbonate/diethyl carbonate (EC/PC/DEC, v:v:v = 1:1:1) containing 2 wt.% fluoroethylene carbonate (FEC) as the electrolyte. Galvanostatic charge–discharge tests were performed at 7.5 mA g^-1^ within a voltage window of 4.5–3.5 V vs. Na⁺/Na (WBCS3000, WonaTech Co.) at room temperature. For full-cell measurements, hard-carbon (HC) anodes were electrochemically pre-sodiated in HC||Na half-cells by cycling at 7.5 mA g^-1^ between 2.0 and 0.005 V for one and a half cycles, ending in the sodiated state. The cells were then disassembled in an Ar-filled glovebox, and the pre-sodiated HC electrodes were used to assemble NMO||HC full cells with an N/P capacity ratio of approximately 1.3. The full cells were cycled between 1.5 and 4.5 V at 7.5 mA g^-1^, and the specific capacities were calculated based on the mass of active NMO material. For in-situ differential electrochemical mass spectrometry (DEMS), the evolution of O_2_ during charge/discharge was monitored using a quadrupole mass spectrometer (HPR-20 R&D, *Hiden Analytical*) with Ar as the carrier gas. The electrochemical cell was directly connected to the mass spectrometer through a capillary inlet to allow real-time gas analysis during cycling.

Galvanostatic intermittent titration technique (GITT) measurements were performed by applying a current pulse of 7.5 mA g^-1^ for 30 min followed by a 2 h relaxation period over the voltage range of 3.5–4.5 V. Electrochemical impedance spectroscopy (EIS) was measured after each relaxation step using a perturbation amplitude of 10 mV over a frequency range of 10^4^–10^-2^ Hz. The acquired impedance spectra were converted into the distribution of relaxation times (DRT) using the DRTtools software package to separate electrochemical processes according to their characteristic relaxation times.^[3]^ The diffusion-related resistance was quantified by integrating the low-frequency DRT response corresponding to the Warburg diffusion region, while the cathode interfacial resistance was obtained by integrating the intermediate relaxation-time region associated with the CEI and charge-transfer processes.

The apparent Na-ion diffusion coefficient (D_Na+_) was estimated from the low-frequency Warburg region by plotting the real part of the impedance Z' (Re) against ω^-1/2^ and fitting the linear region between 0.2 and 0.01 Hz according to Re = R_el_ + R_CEI/ct_ + σω^-1/2^, where R_el_ is the electrolyte resistance, R_CEI/ct_ represents the combined cathode-electrolyte interphase and charge-transfer resistance, σ is the Warburg coefficient, and ω is the angular frequency.^[4]^ The apparent Na-ion diffusion coefficient was calculated using D_Na+_ = $\frac{R^{2}T^{2}}{2A^{2}n^{4}F^{4}C^{2}\sigma^{2}}$ , where (R) is the gas constant, (T) is the absolute temperature, (A) is the electrode area, (n) is the number of electrons transferred, (F) is the Faraday constant, (C) is the Na-ion concentration, and σ is the Warburg coefficient.

*Materials characterization*

Soft X-ray absorption spectroscopy (sXAS) at the O K-edge and X-ray powder diffraction (XPD) were performed at the 23-ID-2 and 28-ID-2 beamlines, respectively, of the National Synchrotron Light Source II (NSLS-II), Brookhaven National Laboratory. Hard X-ray absorption spectroscopy (XAS) at the Mn K-edge was carried out at the 10C beamline of the Pohang Accelerator Laboratory (PAL). All samples were sealed between Kapton tapes in an Ar-filled glove box to prevent air exposure during measurements. Weight changes during the solid-state synthesis of NMO were measured using a thermogravimetric analyzer (TGA2, *Mettler Toledo*) under flowing O₂ with controlled heating and cooling rates matching the synthesis conditions. The morphologies and particle sizes of the NMOs were examined by scanning electron microscopy (Inspect F50) and high-resolution transmission electron microscopy (HR-TEM) (JEM-2010, *JEOL*).

*Computational methods*

Density functional theory (DFT) calculations were performed using the Vienna Ab Initio Simulation Package (VASP),^[5]^ with a plane-wave basis set and projector-augmented wave (PAW) pseudopotentials.^[6]^ The exchange–correlation effects were described by the generalized gradient approximation (GGA), specifically the Perdew–Burke–Ernzerhof (PBE) functional,^[7]^ under spin-polarized conditions. To properly capture the strong on-site Coulomb interactions of the Mn 3d electrons, the GGA+U approach was adopted, with an effective Hubbard U parameter of 3.8 eV for Mn. The plane-wave energy cutoff was set at 520 eV. The convergence criteria were set to 10⁻⁵ eV for electronic self-consistency and 0.03 eV/Å for ionic relaxation. Brillouin zone sampling was carried out using a 1 × 3 × 1 Γ-centered k-point grid. Van der Waals interactions were included via the DFT-D3 dispersion correction scheme.^[8]^ The migration behavior of Mn atoms was investigated using the climbing image nudged elastic band (CI-NEB) method, where intermediate images were fully relaxed until the residual forces on each atom were less than 0.05 eV/Å.

| P$\bar{1}$ | **a (Å)** | **b (Å)** | **c (Å)** | **α (^o^)** | **β (^o^)** | **γ (^o^)** |
| --- | --- | --- | --- | --- | --- | --- |
| **NMO-Q** | **6.5784** | **6.8074** | **7.5527** | **105.1314** | **107.0973** | **112.2315** |
| **NMO-S** | **6.5767** | **6.8262** | **7.5436** | **105.0260** | **107.1054** | **112.2974** |

**Table S1.** Crystallographic parameters for P$\bar{1}$ (P′3) of NMOs refined by profile matching.

| **R3m** | **a (Å) = b** | **c (Å)** | **α (^o^) = β** | **γ (^o^)** |
| --- | --- | --- | --- | --- |
| **NMO-Q** | **2.8015** | **16.1703** | **90** | **120** |
| **NMO-S** | **2.7986** | **16.1565** | **90** | **120** |

**Table S2.** Crystallographic parameters for R3m (P3) phase of NMOs refined by profile matching.

**Fig. S1**. Representative profile matching of SXRD patterns for (a) NMO-S and (b) NMO-Q, showing observed (Y_obs_), calculated (Y_calc_), and difference (Y_obs_−Y_calc_) curves with Bragg reflection positions.

**Fig. S2**. Cyclic performance of (a) NMO-Q (b) NMO-S; Galvanostatic charge/discharge were conducted at 7.5 mA g^-1^ in the range of 4.7−1.5 V_Na+/Na_.

**Fig. S3**. Electrochemical evaluation of O-redox stability of NMOs. Charge/discharge profiles at the initial cycles (upper figures) and second, 5^th^, 15^th^, and 30^th^ cycles (lower figures); Galvanostatic charge/discharge were conducted at 7.5 mA g^-1^ in the range of 4.7−3.5 V_Na+/Na_.

**Fig. S4**. SEM images of NMOs; NMO-S is cooled at a rate of 1.8 ^o^C min^-1^ after isothermal treatment (600 ^o^C for 4 h) and NMO-Q is quenched by immediately dipping the NMO pellet into liquid N_2_ after identical isothermal treatment.

**Fig. S5.** Charge–discharge profiles of three independently assembled (a) NMO-Q and (b) NMO-S cells. Scatter plots comparing the corresponding (c) 2^nd^ cycle discharge capacities and (d) 2^nd^ cycle voltage hysteresis values. Galvanostatic charge/discharge were conducted at 7.5 mA g^-1^ in the range of 4.5−3.5 V_Na+/Na_. The datasets include the cells presented in Figs. 2a and 2c of the main text.

**Fig. S6.** Cyclic performance of (a) NMO-Q and (b) NMO-S; Galvanostatic charge/discharge were conducted at 7.5 mA g^-1^ in the range of 4.5−3.5 V_Na+/Na_.

**Fig. S7.** Electrochemical evaluation of O-redox stability of NMOs. Charge/discharge profiles at the initial cycles (upper figures) and second, 5^th^, 15^th^, and 30^th^ cycles (lower figures); Galvanostatic charge/discharge were conducted at 7.5 mA g^-1^ in the range of 4.3−3.5 V_Na+/Na_.

**Fig. S8**. Cyclic performance of (a) NMO-Q (b) NMO-S; Galvanostatic charge/discharge were conducted at 7.5 mA g^-1^ in the range of 4.3−3.5 V_Na+/Na_.

**Fig. S9**. Cyclic performance of (a) NMO-Q (b) NMO-S; Galvanostatic charge/discharge were conducted at 7.5 mA g^-1^ in the range of 4.7−3.5 V_Na+/Na_.

**Fig. S10.** Rate performance of NMO-Q and NMO-S measured at current densities of 7.5, 15, 30, 75, and 150 mA g^-1^ within the voltage range of 4.5–3.5 V.

**Fig. S11.** Combined GITT and EIS measurements of NMO-Q and NMO-S. GITT profiles of (a) NMO-Q and (b) NMO-S during the first charge/discharge cycle. Corresponding EIS Nyquist plots collected after each relaxation step for (c) NMO-Q and (d) NMO-S. A current pulse of 7.5 mA g^-1^ was applied for 30 min, followed by a 2 h relaxation period. EIS spectra were collected after each relaxation step over the frequency range of 10^4^ – 10^-2^ Hz with a 10 mV perturbation.

**Fig. S12.** Distribution of relaxation times (DRT) analysis of the EIS spectra shown in Fig. S11. DRT profiles of (a) NMO-Q and (b) NMO-S during the first charge/discharge cycle. Evolution of the diffusion-related resistance, cathode interfacial resistance (including CEI and charge-transfer contributions), and total resistance for (c) NMO-Q and (d) NMO-S. Apparent Na-ion diffusion coefficients calculated from the Warburg coefficients obtained by linear fitting in the 0.2–0.01 Hz frequency range for (e) NMO-Q and (f) NMO-S.

**Fig. S13**. Line plots of TR-XRD patterns with times during heating treatment of precursor mixture (NaNO_3_ and MnCO_3_, 2:3 in molar ratio); The mixture was heated to 600 ^o^C at a heating rate of 5 oC min^-1^.

**Fig. S14**. Line plots of TR-XRD patterns with times during heating treatment of precursor mixture (NaNO_3_ and MnCO_3_, 2:3 in molar ratio); The mixture was heated at 600 ^o^C for 4 h at a heating rate of 5 ^o^C min^-1^.

**Fig. S15**. Line plots of TR-XRD patterns collected during the cooling process for a mixture of NaNO_3_ and MnCO_3_ (molar ratio = 2:3): (a) from 600 to 520 ^o^C; (b) from 520 to 280 ^o^C; (c) from 280 to 50 ^o^C. (d) Comparison of XRD patterns obtained at 600, 520, and 420 ^o^C. The precursor mixture was heated at 600 ^o^C for 4 h with a heating rate of 5 ^o^C min^-1^ and subsequently cooled at a rate of 1.8 ^o^C min^-1^. The resulting sample is denoted as NMO-S in this work

**Fig. S16**. Crystallographic visualization of in-plane Mn/□_Mn_ ordering in P′3-Na_2_Mn_3_O_7_. Purple octahedra represent MnO_6_ units and yellow spheres denote Na⁺ ions; the magenta planes highlight the (001), (11-1), (-102), and (101) lattice planes associated with the √7×√7 Mn/□Mn ordering.

**Fig. S17.** Repeated temperature-resolved XRD measurement of NMO-S using an independently prepared precursor batch. (a) Contour plots of XRD patterns with times during heating and isothermal treatment of precursor mixture (NaNO_3_ and MnCO_3_). (b) Enlarged contour plots of (a) in the range of 12.0 − 14.5 ^o^ and 16.5 − 30.0 ^o^. (c) TR-XRD patterns of NMO-S collected during cooling process (cooling rate: 1.8 ^o^C min^-1^).

**Fig. S18**. (a) TEM image of NMO-S and (b) synchrotron-based X-ray pair distribution function (PDF) results.

**Fig. S19**. Time-resolved X-ray diffraction (TR-XRD) patterns during synthesis of NMO (a) Contour plots of TR-XRD patterns with times during cooling process (cooling rate: 9.0 ^o^C min^-1^); (b) Enlarged contour plots in the range of 12.0 − 14.5 ^o^ and 16.5 − 30.0 ^o^. The mixture of NaNO_3_ and MnCO_3_ (2:3 in molar ratio) were heated at 600 ^o^C for 4 h at a heating rate of 5 ^o^C min^-1^.

**Fig. S20**. TGA result corresponding to Fig. S19.

**Fig. S21**. Charge/discharge profiles of NMO cooled at 9 °C min⁻¹ (corresponding to Figs. S19 and S20). Galvanostatic charge/discharge were conducted at 7.5 mA g⁻¹ in the range of 4.5–3.5  V_Na+/Na_.

**Fig. S22.** O K-edge of NMOs at pristine, first charged, and discharged states. (a) NMO-Q (b) NMO-S. Galvanostatic charge/discharge were conducted at 7.5 mA g^-1^ in the range of 4.5−3.5 V_Na+/Na_.

**Fig. S23**. In-situ XRD analysis of (a) NMO-S and (b) NMO-Q during initial charge and discharge. Galvanostatic charge/discharge were conducted at 7.5 mA g^-1^ in the range of 4.7−3.5 V_Na+/Na_. Corresponding sequential Le Bail refinement results showing the relative changes in (c) a/a_0_, (d) b/b_0_, (e) c/c_0_, and (f) (V-V_0_)/V_0_ during the first and second charge–discharge cycles.

**Fig. S24**. In-situ XRD analysis for the superstructure region of (a) NMO-S and (b) NMO-Q during initial charge discharge. Galvanostatic charge/discharge were conducted at 7.5 mA g^-1^ in the range of 4.7−3.5 V_Na+/Na_.

**Fig. S25.** Charge/discharge profiles of (a) NMO-Q||hard-carbon and (b) NMO-S||hard-carbon full cells during the first cycle. The cells were assembled using pre-sodiated hard-carbon anodes with an N/P ratio of approximately 1.3 and tested between 1.5 and 4.5 V at 7.5 mA g^-1^. Specific capacities were calculated based on the mass of active NMO material.

**References**

[1] D. H. Kim, G. Ali, J.-Y. Kim, K.-B. Kim, K. Y. Chung, “Real-time X-ray analytical micro-furnace technique for in situ phase formation analysis during material synthesis,” *Energy Storage Materials* 59 (2023): 102766, <https://doi.org/10.1016/j.ensm.2023.04.005>.

[2] D. H. Kim, J.-Y. Kim, J.-H. Park, S.-O. Kim, H.-S. Kim, K.-B. Kim, K. Y. Chung, “RT-XAMF and TR-XRD studies of solid-state synthesis and thermal stability of NaNiO2 as cathode material for sodium-ion batteries,” *Ceramics International* 48 (2022): 19675–19680, <https://doi.org/10.1016/j.ceramint.2022.03.104>.

[3] T. H. Wan, M. Saccoccio, C. Chen, F. Ciucci, “Influence of the Discretization Methods on the Distribution of Relaxation Times Deconvolution: Implementing Radial Basis Functions with DRTtools,” *Electrochimica Acta* 184 (2015): 483–499, <https://doi.org/10.1016/j.electacta.2015.09.097>.

[4] H. B. Smith, G.-H. Lee, B. S. Kumar, A. N. Penn, V. Venturi, Y. Gao, R. C. Davis, K. H. Stone, A. Hunt, I. Waluyo, E. Stavitski, W. Yang, I. I. Abate, “Elucidating the effect of Fe substitution on structural and redox stability of Na2Mn3O7,” *Journal of Materials Chemistry A* 13 (2025): 11466–11474, <https://doi.org/10.1039/D4TA08203F>.

[5] G. Kresse, J. Furthmüller, “Efficient iterative schemes for ab initio total-energy calculations using a plane-wave basis set,” *Physical Review B* 54 (1996): 11169–11186, <https://doi.org/10.1103/PhysRevB.54.11169>.

[6] P. E. Blöchl, “Projector augmented-wave method,” *Physical Review B* 50 (1994): 17953–17979, <https://doi.org/10.1103/PhysRevB.50.17953>.

[7] J. P. Perdew, K. Burke, M. Ernzerhof, “Generalized Gradient Approximation Made Simple,” *Physical Review Letters* 77 (1996): 3865–3868, <https://doi.org/10.1103/PhysRevLett.77.3865>.

[8] S. Grimme, J. Antony, S. Ehrlich, H. Krieg, “A consistent and accurate ab initio parametrization of density functional dispersion correction (DFT-D) for the 94 elements H-Pu,” *The Journal of Chemical Physics* 132 (2010): 154104, <https://doi.org/10.1063/1.3382344>.
